# Willingness to Receive Maternal RSV Vaccination Among Pregnant Women and Those Planning Pregnancy in Southern China: A Cross-Sectional Study and Predictive Nomogram

**DOI:** 10.3390/vaccines14020160

**Published:** 2026-02-08

**Authors:** Xiang Meng, Sijie Li, Meiyan Li, Cheng Guo, Ping Wang, Xuejuan Chen, Dingmei Zhang, Yonghui Zhong

**Affiliations:** 1 Department of Epidemiology, School of Public Health, Sun Yat-Sen University, No. 74, Zhongshan 2nd Road, Yuexiu District, Guangzhou 510080, China; mengx8@mail2.sysu.edu.cn (X.M.); lisj63@mail2.sysu.edu.cn (S.L.); guoch36@mail.sysu.edu.cn (C.G.); 17512996990@163.com (X.C.); 2 Tuberculosis Department, Foshan Fourth People’s Hospital, No. 106, Jinlan South Road, Chancheng District, Foshan 528000, China; drlimeiyan88@126.com; 3 Guangzhou Women and Children’s Medical Center, Guangzhou Medical University, No. 9, Jinsui Road, Tianhe District, Guangzhou 510623, China; wangping486@126.com

**Keywords:** respiratory syncytial virus, maternal vaccination, vaccine willingness, pregnancy, nomogram

## Abstract

Background/Objectives: Maternal immunization against respiratory syncytial virus (RSV) is an emerging strategy to protect infants during early life when they are most vulnerable to severe RSV infection. However, little is known about the willingness to receive maternal RSV vaccination in China, where the vaccine has not yet been officially approved for marketing. This study aimed to assess the willingness to receive maternal RSV vaccination among women who are currently pregnant and those planning pregnancy in Guangzhou, and to identify the key determinants influencing vaccination willingness. Methods: A cross-sectional survey was conducted in April 2025 among 406 women at Guangzhou Women and Children’s Medical Center, China. Participants completed a self-administered questionnaire covering predisposing factors, enabling resources, health behaviors and awareness, and need factors. Logistic regression analyses were used to identify factors associated with vaccine willingness. A nomogram prediction model was constructed based on significant predictors. Results: Overall, 67.2% (*n* = 273) of participants reported willingness to receive maternal RSV vaccination. Younger maternal age, higher levels of social support, moderate or high perceived RSV risk, a history of HPV vaccination, and having medical insurance were independently associated with higher willingness to vaccinate. A predictive nomogram incorporating these factors demonstrated good discrimination (AUC = 0.753) and calibration. Age-stratified analysis revealed differing concerns across age groups, with vaccine safety and neonatal protection being the most cited factors influencing decision-making. Conclusions: This study provides the first evidence on maternal RSV vaccination willingness in southern China and highlights several psychosocial and demographic factors influencing vaccine intentions. The nomogram offers a practical tool to estimate individual willingness and guide targeted communication. These findings have implications for future maternal RSV vaccine application strategies in China.

## 1. Introduction

Respiratory syncytial virus (RSV) is a leading cause of acute respiratory tract infections in infants and young children. It is estimated that approximately 90% of children under the age of two have experienced at least one RSV infection, with the peak incidence typically occurring between 6 and 12 months of age [1,2,3]. Infants under one year of age are particularly vulnerable, exhibiting both higher infection rates and an increased risk of developing serious lower respiratory tract complications [4,5,6].

RSV possesses two major surface glycoproteins—*G* protein and fusion (*F*) protein—both of which can induce protective neutralizing antibody responses [7]. The *F* protein is highly conserved between the two RSV subtypes, with approximately 90% sequence identity, making it a particularly promising target for vaccine development [8]. Notably, the prefusion conformation of the *F* protein (pre-*F*) retains a greater number of neutralization-sensitive epitopes compared to the post fusion form and is recognized as the major target of RSV-neutralizing antibodies in human sera [9]. During RSV infection, anti-RSV IgG antibodies primarily act by binding to these pre-*F*-specific epitopes, thereby blocking viral fusion with host cells and conferring protection [10,11,12].

Given the immaturity of the neonatal immune system, protection against RSV infection in early infancy largely relies on maternally derived neutralizing antibodies transferred transplacentally during pregnancy [11,13,14]. Accordingly, maternal immunization during pregnancy has emerged as a promising strategy to provide passive immunity and protect newborns during the critical early period of high RSV susceptibility. In August 2023, the U.S. Food and Drug Administration (FDA) approved an RSV vaccine developed by Pfizer for use in pregnant women between 24 and 36 weeks of gestation [15]. This vaccine has since been approved in several other countries and regions, including Canada, the European Union, the United Kingdom, Japan, and Australia [16]. However, it is not yet approved for use in China.

In China, the burden of RSV-related illness in infants and young children remains substantial, yet public awareness of RSV and its prevention is limited. Effective prophylactic options are also scarce. Currently, the only approved intervention in China for preventing RSV in infants is Nirsevimab, a long-acting monoclonal antibody [17,18]. While monoclonal antibodies offer passive protection, maternal vaccination represents a potentially more scalable preventive strategy, particularly for infants who are too young or medically ineligible to receive direct immunization [19]. By boosting maternal antibody levels, maternal vaccination can confer indirect protection to newborns and may also contribute to population-level protection by reducing RSV transmission in high-risk settings [20,21,22].

Despite the substantial disease burden and the promise of maternal immunization, awareness of RSV among mothers remains low globally, and evidence from low- and middle-income settings highlights persistent knowledge gaps. For example, in The Gambia, only 12.8% of mothers had heard of RSV, and merely 10.6% recognized its potential to cause life-threatening illness in infants [23]. Beyond awareness, maternal decision-making regarding vaccination is shaped by a range of sociodemographic, experiential, and contextual factors. Mothers who have experienced RSV infection in their children often face considerable emotional and financial stress, which may influence their openness to preventive measures such as vaccination [24]. Prior studies have shown that education level, age, and previous vaccination experience are important predictors of vaccine acceptance. In Jordan, higher acceptance was observed among mothers with undergraduate education and those working in healthcare [25]. Moreover, recommendations from healthcare providers consistently emerge as a key facilitator of vaccine uptake, as demonstrated in studies from both The Gambia and Italy, whereas concerns about vaccine safety and availability remain prominent barriers [23,26].

Although previous studies have identified various psychosocial factors—such as knowledge, risk perception, and social support—that influence willingness to receive vaccination, data on the willingness of currently pregnant women and those planning pregnancy in China to receive RSV vaccination are lacking [27,28,29]. Therefore, before introducing maternal RSV vaccination in China, it is essential to understand the willingness of the target population and to identify key determinants of vaccination willingness. This information is critical for informing public health strategies and optimizing vaccine implementation policies.

## 2. Materials and Methods

### 2.1. Study Design and Participants

This cross-sectional study was carried out using the convenience sampling method. In April 2025, we distributed our self-developed questionnaire (Supplementary Materials) on-site at the outpatient clinic of Guangzhou Women and Children’s Medical Center, waited at the survey site, and collected the completed questionnaires on the spot. All questionnaires were self-administered, and participation was voluntary and anonymous; participants could decline any item or withdraw at any time. Participants were women who were either preparing for pregnancy or were currently pregnant during the study period.

The sample size was estimated based on the formula adapted for the cross-sectional study:

$$n\;=\;\frac{Z_{\alpha /2}^{2}\;\times \;p\;\times \;(1-p)}{d^{2}}$$

The threshold of α and precision were set at 0.05, and Z_α/2_ was 1.96. Based on our preliminary survey, the proportion (expected proportion of willingness to vaccinate) was 71.9%. Therefore, the minimum sample size was 310. During the survey period, a total of 406 questionnaires were collected. The participants in this study were enlisted without any financial compensation to ensure the validity of the findings. Due to on-site administration and immediate completeness checks, no missing data were present in the final analytic dataset; therefore, imputation was not performed.

This study was reviewed and approved by the Biomedical Research Ethics Committee of the School of Public Health, Sun Yat-sen University. Given the minimal-risk and questionnaire-based design of the study, written informed consent was not required by the ethics committee. Instead, oral informed consent was obtained from all participants after a clear explanation of the study objectives, procedures, voluntary nature of participation, and data confidentiality. Only individuals who agreed to participate were invited to complete the questionnaire; those who declined were not included in the study. All data were collected anonymously and used solely for research purposes.

### 2.2. Willingness to Receive Maternal RSV Vaccination

Willingness to receive maternal RSV vaccination was the primary outcome of interest. It was assessed using a single questionnaire item (Q34) asking participants whether they would be willing to receive an RSV vaccine during pregnancy if it were recommended by national immunization guidelines or healthcare providers. Responses were recorded as “Willing” or “Unwilling” and treated as a binary outcome variable in all analyses. This item was designed to capture respondents’ stated vaccination intention under a standardized recommendation scenario, thereby reducing variability related to vaccine availability or policy uncertainty at the time of the survey.

### 2.3. Influencing Factors

A total of 33 potential influencing factors were assessed based on a structured questionnaire informed by Andersen’s Behavioral Model of Health Services Use [30], which posits that health-related behaviors are shaped by three domains: predisposing factors (e.g., age, education), enabling resources (e.g., income, insurance, social support), and perceived needs (e.g., health status, perceived risk). Based on this framework, we conceptualized health-related behaviors as the result of interactions among predisposing factors, enabling resources, health behaviors and awareness, and need factors. We hypothesized that maternal willingness to receive RSV vaccination during pregnancy would be influenced by multiple domains. Specifically, we hypothesized that: (1) predisposing factors such as younger maternal age and pregnancy status would be associated with higher vaccination willingness; (2) enabling resources, including higher social support, medical insurance coverage, and higher household income, would be positively associated with vaccination willingness; (3) health behaviors and awareness, such as prior awareness of RSV, higher perceived RSV risk, greater information availability, higher RSV knowledge level, and previous vaccination history, would be associated with increased willingness; and (4) need-related factors reflecting perceived or evaluated health needs would further influence maternal vaccination intention. All influencing factors were collected at baseline via self-administered questionnaires and grouped into four conceptual domains [31].

#### 2.3.1. Predisposing Factors

These included basic sociodemographic and reproductive characteristics: maternal age (Q2), ethnicity (Q3), religion (Q4), education level (Q5), occupation (Q6), residence type (Q7–Q8), marital status (Q9), co-residing conditions (Q10), parity (Q11, excluding the current pregnancy), and pregnancy status (Q12, planning pregnancy or in the first, second, or third trimester).

#### 2.3.2. Enabling Resources

Enabling factors comprised both financial and social resources. Financial variables included household annual income (Q13), low-income household status (Q14) and usual type of healthcare facility (Q16). Social support from family members, friends, and healthcare professionals was measured using a 5-point Likert scale, with higher scores indicating greater perceived social support (Q15) [32].

#### 2.3.3. Health Behaviors and Awareness

This domain included RSV-related information sources, prior awareness of RSV, RSV knowledge level, and vaccination history. RSV knowledge was assessed using six true/false items and summarized as a knowledge score ranging from 0 to 6 (Q19–Q24); participants were categorized into high or low knowledge groups using the median score as the cutoff. Prior awareness and risk perception were measured using Likert-scale items and categorized into low, moderate, or high levels based on quartile-based cutoffs (Q25–Q27). Previous vaccination history included self-reported receipt of selected recommended vaccines and vaccinations during pregnancy (Q28–29).

#### 2.3.4. Need-Related Factors

Need-related factors captured respondents’ perceived or evaluated health needs, including the presence of chronic conditions (Q30), specific medical contraindications or histories that could influence vaccination decisions (Q31), medical insurance programs (Q32) and selected factors reflecting perceived susceptibility or concern related to maternal or infant health (Q33).

The definitions, measurement methods, categorization, and coding of all variables included in the analysis are summarized in Table S1. The content of the questionnaire was reviewed and approved by professors and domain experts before formal distribution. Reliability analysis indicated that the Cronbach’s α values for the social support scale and the risk perception scale were 0.90 and 0.76, respectively, both exceeding the commonly accepted threshold of 0.70, suggesting good internal consistency [33].

Prior to field administration, we conducted a small pilot test (30 respondents) via an online platform to assess item clarity and completion time. Minor wording and layout refinements were made; the instrument’s structure and scoring rules were unchanged. Because the content remained identical, eligible pilot responses were retained in the final analytic sample under the same inclusion criteria.

### 2.4. Statistical Analysis

#### 2.4.1. Descriptive Analysis

All statistical analyses were performed using R software (version 4.3.3; R Foundation for Statistical Computing, Vienna, Austria). Descriptive statistics were used to summarize participants’ characteristics, with continuous and categorical variables presented as mean ± standard deviation and frequency (percentage, %), and compared by chi-squared test or Fisher’s exact test.

#### 2.4.2. Binary Logistic Regression

Binary logistic regression analyses were conducted in accordance with the research model described above to examine the associations between conceptually defined predictors and maternal RSV vaccination willingness. We initially conducted univariable logistic regression to explore crude associations between potential predictors and vaccination willingness (Table S3).

Variables were selected for inclusion in the multivariate logistic regression model based on their theoretical relevance within the predefined research framework, evidence from prior literature, and exploratory group comparisons or univariable analyses (*p* < 0.1) [34]. Variables representing each conceptual domain were retained in multivariable analyses to ensure theoretical coherence and to avoid data-driven selection based solely on statistical significance. The final multivariable model included the following 11 variables: maternal age, pregnancy status, annual household income, social support level, prior awareness of RSV (heard of RSV), perceived information availability regarding RSV, RSV knowledge level, perceived RSV risk, history of Hepatitis B vaccination, history of HPV vaccination, and medical insurance status. The specific categorization and reference group definitions for each independent variable are detailed in Table S1. The dependent variable in the multivariable logistic regression was maternal willingness to receive RSV vaccination during pregnancy, defined as a binary outcome (willing vs. unwilling).

These variables were selected to represent the four conceptual components specified in the questionnaire framework. Specifically, maternal age and pregnancy status were treated as predisposing factors; annual household income, social support level, and medical insurance status were considered enabling resources; prior awareness of RSV, perceived information availability, RSV knowledge level, perceived RSV risk, and prior vaccination history (Hepatitis B and HPV) were categorized as health behaviors and awareness; and variables reflecting medical conditions or contraindications were assessed as needing factors.

To reduce the risk of omitting conceptually important predictors and to ensure adequate adjustment for key socioeconomic and cognitive factors relevant to vaccination decision-making, annual household income and RSV knowledge level were retained in the multivariable model despite not meeting the univariable screening threshold. To minimize overfitting, model complexity was constrained to maintain at least approximately 10 events per predictor variable (EPV). Odds ratios (ORs) and 95% confidence intervals (CIs) were calculated to estimate associations with vaccination willingness, with statistical significance defined as a two-sided *p* < 0.05. For continuous variables (e.g., maternal age), ORs represent the change in odds per one-unit increase.

Multicollinearity among predictors was assessed using variance inflation factors (VIF/GVIF). All adjusted GVIF values (GVIF^(1/(2×f))) were low (range: 1.03–1.18), indicating no concerning multicollinearity. Model calibration was evaluated using the Hosmer–Lemeshow goodness-of-fit test (χ^2^ = 8.36, df = 8, *p* = 0.399), suggesting adequate model fit. Model discrimination was assessed using the area under the receiver operating characteristic curve (AUC = 0.753). In addition, we examined the linearity-in-the-logit assumption for the continuous predictor (maternal age) and screened for influential observations, and no major violations were identified.

#### 2.4.3. Nomogram Prediction

A multivariable logistic regression-based nomogram was constructed to visually depict the predicted probability of maternal RSV vaccination willingness based on significant determinants identified in the regression model. The final model included maternal age, social support level, RSV knowledge level, perceived RSV risk, HPV vaccination, and medical insurance status. All continuous predictors, including maternal age, were evaluated for potential non-linear relationships using restricted cubic splines; no substantial non-linearity was detected. The final nomogram was constructed using the *β* coefficients from the multivariable logistic regression model, and point values were assigned using the ‘rms’ package in R.

The nomogram was designed to assign point values to each level of the included predictors, with the total points summing to an estimated probability of willingness to vaccinate. The nomogram function with a logistic transformation (plogis) was used to calibrate predicted probabilities. Internal validation was performed using 1000 bootstrap resamples to assess model performance. Discrimination was quantified using the area under the receiver operating characteristic curve (AUC), and calibration was evaluated by plotting predicted versus observed probabilities. Decision curve analysis was also performed to evaluate clinical utility. The mean absolute error of the calibration curve was 0.014. To enhance interpretability, we overlaid the nomogram with kernel density plots of variable distributions and highlighted a sample case to illustrate practical application.

## 3. Results

### 3.1. Descriptive Statistics for Participants

Among the 406 participants included in the final analysis, 67.2% reported willingness to receive the RSV vaccine during pregnancy. As shown in descriptive analysis (Table 1), women who expressed willingness to vaccinate (30.00 ± 4.28 years) were significantly younger on average compared to those who were unwilling (31.24 ± 4.29 years). Pregnancy status was also associated with vaccination intention, with a higher proportion of women in the later stages of pregnancy indicating willingness to receive the vaccine. Participants with higher levels of perceived social support, a higher perceived risk of RSV, having previously heard of RSV and perceiving that information on RSV was sufficiently available, were significantly more likely to report positive vaccination intentions. Vaccination history was also a distinguishing factor: those who had previously received hepatitis B or HPV vaccines showed significantly greater willingness. Moreover, women with any type of medical insurance were more likely to express an intention to receive the RSV vaccine than those without.

### 3.2. Binary Regression of Influencing Factors of Vaccination Intention

In univariate logistic regression analyses, several factors were significantly associated with vaccination willingness, including maternal age, pregnancy status, annual household income, perceived social support level, heard of RSV, information availability, perceived RSV risk, history of vaccination, and medical insurance coverage.

In the multivariable logistic regression analysis (Table 2), maternal age was negatively associated with vaccination intention (*β* = 0.06, 95% CI: −0.12–−0.01, *p* = 0.035). Higher levels of perceived social support were strongly associated with increased willingness to receive the RSV vaccine (OR = 3.85, 95% CI: 2.33–6.46, *p* < 0.001). Perceived RSV risk was another significant predictor: compared to women with low perceived risk, those with moderate (OR = 2.23, 95% CI: 1.32–3.81, *p* = 0.003) or higher risk perception (OR = 2.12, 95% CI: 1.03–4.51, *p* = 0.045) were more likely to express willingness to vaccinate. Women who had received the HPV vaccine were also more likely to express willingness to receive the RSV vaccine (OR = 1.77, 95% CI: 1.07–2.95, *p* = 0.026), while the influence of the Hepatitis B vaccine turned insignificant. Lastly, the coverage of medical insurance was associated with a greater willingness to vaccinate (OR = 3.77, 95% CI: 1.17–12.86, *p* = 0.028).

### 3.3. Nomogram Prediction of RSV Vaccination Willingness

**Figure 1:**
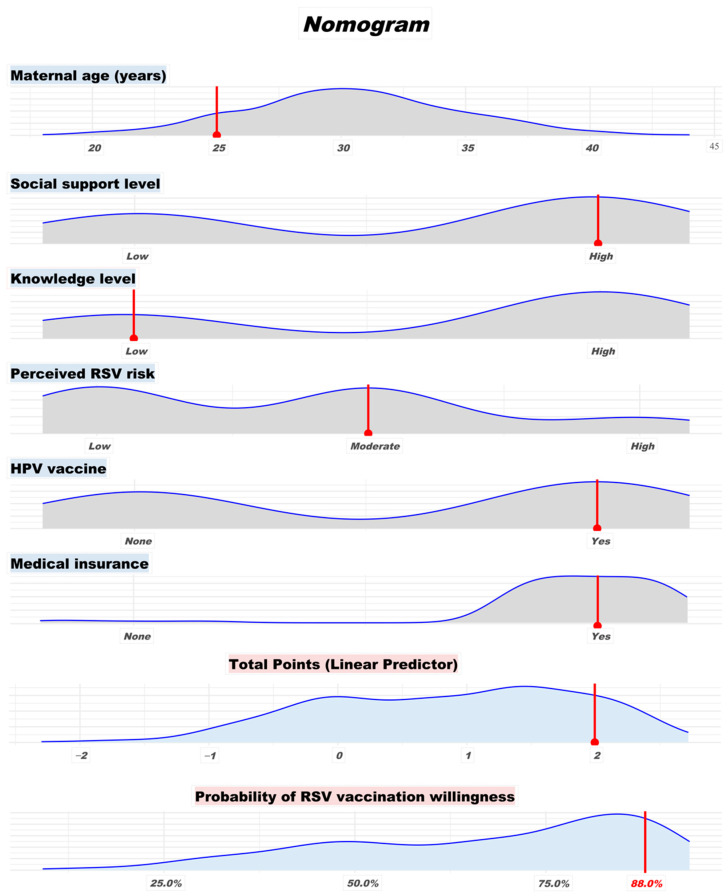
Predictive nomogram for maternal RSV vaccination willingness. Each predictor is plotted with a corresponding distribution, and red markers indicate an individual participant’s profile. The total score is translated into a predicted probability of RSV vaccination willingness. This represents an absolute predicted probability (not a relative risk), facilitating threshold-based clinical counseling and shared decision-making. Blue lines indicate variable distribution density, gray areas reflect population frequency, and red dots/lines represent the representative example prediction.

Based on the final multivariate logistic regression model, a predictive nomogram was developed to estimate the probability of RSV vaccination willingness. As illustrated in Figure 1, each predictor contributes a distinct point score corresponding to its impact on vaccination intention. For instance, younger maternal age, higher social support level, moderate-to-high perceived RSV risk, prior HPV vaccination, and having medical insurance were all associated with increased predicted probability. The total point sum was mapped to a probability scale, which allowed intuitive estimation of an individual’s probability of willingness to receive vaccination. The nomogram was further enhanced by incorporating variable-specific density plots and marking a sample case, providing both visual interpretability and clinical utility. The nomogram outputs an absolute predicted probability of vaccination willingness (0–1/0–100%) derived from the logistic model, which can be paired with threshold-based counseling during antenatal visits.

To illustrate practical application, we applied the nomogram to a representative participant—a 25-year-old woman with high social support, low RSV knowledge, moderate perceived RSV risk, a history of HPV vaccination, and current medical insurance coverage. Based on these characteristics, her total point score corresponded to a predicted RSV vaccination willingness probability of 88.0%, as indicated by the red markers on the nomogram. In the nomogram, red dashed lines represent reference guides that project each predictor to a point scale, and the total score is then mapped to a predicted probability of vaccination willingness, as derived from the logistic regression model. This nomogram is intended for visual and educational purposes. For practical application, a corresponding scoring table summarizing point assignment for each variable level is provided in Table S4.

The nomogram demonstrated good discrimination ability, with an area under the receiver operating characteristic (ROC) curve (AUC) of 0.753, indicating a moderate capacity to distinguish between women with high versus low vaccination intention (Figure S1). Internal validation based on 1000 bootstrap resamples showed excellent calibration, with strong agreement between predicted and observed probabilities (mean absolute error = 0.014), suggesting that the model was well-calibrated and not overfitting (Figure S2). Furthermore, decision curve analysis (DCA) supported the nomogram’s potential clinical utility for informing individualized recommendations or targeted vaccination strategies (Figure S3).

### 3.4. Age-Stratified Preferences for Vaccination Determinants

**Figure 2:**
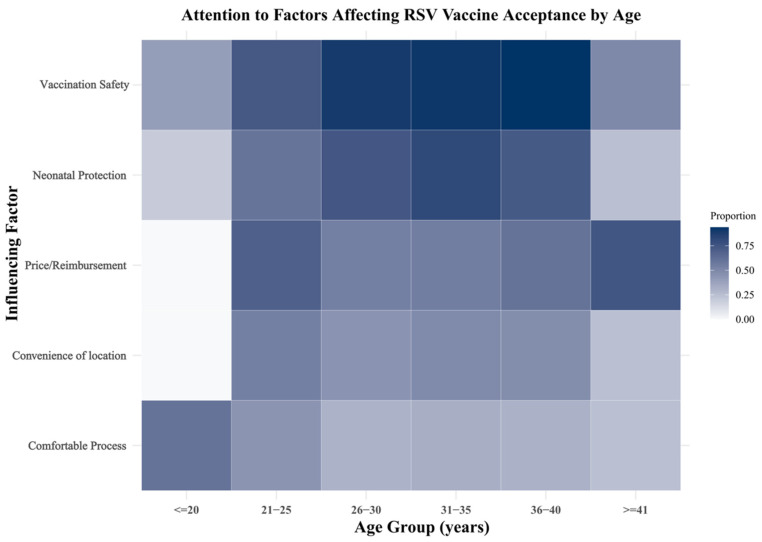
Heatmap of attention to determinants of RSV vaccination willingness by maternal age group. Rows represent candidate factors; columns represent age groups. Cell colors show selection proportions (darker cells = relatively greater attention). Categorical variables were grouped per the questionnaire coding.

We used a heatmap analysis to illustrate the attention to various potential factors influencing RSV vaccination willingness across different maternal age groups (Figure 2), with rows denoting candidate factors and columns denoting maternal age groups. Color intensity reflects the magnitude of the proportion; factors are shown in their pre-specified order. Overall, vaccine safety and neonatal protection were the most frequently selected concerns among all age groups. Women aged 26–40 years showed the highest levels of concern for vaccine safety, while younger women (≤25 years) placed relatively greater emphasis on price and convenience of vaccination.

Older age groups (≥31 years) consistently expressed strong concern about the protective effect of vaccination for their newborns. In contrast, perceived comfort of the vaccination process showed relatively low variation across age groups. These findings suggest that communication strategies to promote RSV vaccination should be tailored to age-specific concerns.

## 4. Discussion

In this study, we identified maternal age, social support level, perceived risk of RSV infection, HPV vaccination status, and medical insurance coverage as factors associated with intention to receive maternal RSV vaccination among women who are currently pregnant or preparing for pregnancy. We developed a nomogram model to predict maternal RSV vaccination willingness. The nomogram demonstrated moderate discrimination (AUC = 0.753), good calibration (mean absolute error = 0.014), and promising clinical utility, suggesting its potential as a visual risk assessment tool. However, further external validation is needed before clinical application.

Globally, studies exploring the willingness of maternal RSV vaccination during pregnancy remain limited. A study conducted in Italy found that higher perceived risk of RSV infection, previous influenza vaccination during pregnancy, and having public insurance were positively associated with maternal RSV vaccination willingness among currently pregnant and preparing for pregnancy women. Our findings extend the relevance of these similar predictors to the Chinese population [28]. Moreover, previous research has demonstrated that confidence in vaccines is also a significant predictor of maternal RSV vaccination [29]. Several studies have consistently highlighted that healthcare provider recommendation plays a crucial role in shaping maternal vaccine decision-making [27,29,35].

Additionally, studies on maternal willingness to other vaccines have shown that factors such as maternal age, knowledge about the disease and the corresponding vaccine, family and social support, and encouragement from healthcare providers are all associated with vaccine uptake during pregnancy. Although these studies did not focus on RSV vaccines specifically, the consistency of these findings lends further support to the relevance of our results [31,32,36,37]. Given China’s unique sociocultural, healthcare, and policy context, localized data are essential for informing national immunization strategies. International evidence on other maternal vaccines also shows wide variability in acceptance, with reported influenza and pertussis vaccination coverage among pregnant women ranging from about 10–20% in some middle-income settings to over 50–60% in high-income countries, and a pooled global intention of approximately 47% for COVID-19 vaccination during pregnancy [38,39,40]. Taken together, these figures suggest that the 67.2% willingness to receive maternal RSV vaccination observed in our sample falls within the mid-range of maternal vaccine acceptance internationally, while still indicating substantial room for improvement through context-specific strategies in China.

In China, family decision-making and trust in healthcare providers are key factors influencing maternal vaccine acceptance. Research shows that in Chinese culture, family members, particularly grandparents, significantly influence health decisions. This family-centric decision-making model can affect mothers’ willingness to accept vaccines, especially when older relatives are skeptical of modern medical practices [41]. Additionally, trust in healthcare providers plays a crucial role. Past vaccine safety incidents and medical scandals have increased mistrust, leading many mothers to hesitate in vaccinating their children [42]. Despite advancements in modern medicine, traditional medicine still holds significant sway in many households, presenting a barrier to vaccine acceptance [43]. Furthermore, gender roles influence vaccine decision-making, as fathers or elders often have the final say in many Chinese families, limiting the mother’s autonomy in health decisions [41]. The rural–urban divide also plays a role, with urban mothers having better access to healthcare information and services, which directly affects vaccine uptake [23].

It is noteworthy that several conventional sociodemographic and clinical characteristics (e.g., education level, household income, place of residence, parity, chronic disease history, usual healthcare facility, and co-residents requiring care) were not associated with maternal RSV vaccination intention. These null associations may reflect attenuation after adjustment for enabling and perceived-need constructs (e.g., social support, perceived risk), limited category variation and statistical power within specific subgroups, and contextual features (e.g., pertussis vaccination is not routinely offered in pregnancy in mainland China). We therefore view these as informative nulls that help prioritize broader measurement in future work (e.g., obstetric histories, prior vaccine adverse events) to reduce residual confounding and improve model specification. This null finding may be partly explained by the relatively homogeneous and socioeconomically advantaged profile of our sample—most participants were urban, highly educated women with near-universal medical insurance recruited from a tertiary maternal health center—which likely attenuated observable socioeconomic gradients in vaccine intention [44]. HPV vaccination, but not routine vaccinations such as influenza, COVID-19, MMR, tetanus, rabies or vaccines received during pregnancy, was associated with RSV vaccine intention, suggesting that self-paid, selective vaccines may better capture a general pro-vaccine orientation than widely recommended or mandatory vaccines. This pattern is also consistent with the possibility that widely recommended vaccines provide less discriminatory variation in this sample, whereas selective, self-paid vaccines capture underlying vaccine confidence more strongly.

As RSV vaccines become increasingly available worldwide, this study provides timely evidence on vaccine intention among pregnant women and those planning pregnancy in China, supporting the development of personalized maternal immunization programs. A visual nomogram model was developed for individualized prediction, offering a practical tool for public health risk stratification and communication. Notably, the model’s performance was internally validated using 1000 bootstrap resamples and evaluated through discrimination, calibration, and decision curve analyses. This study incorporated key psychosocial correlates—such as social support, risk perception, and vaccine knowledge—providing a more realistic and multidimensional framework for assessing vaccine intention. Looking forward, this nomogram could be feasibly integrated into clinical practice to support personalized vaccine counseling. For instance, it may be embedded into electronic prenatal medical records or mobile health (mHealth) applications to help antenatal care providers quickly identify women at higher risk of vaccine hesitancy and tailor communication accordingly. Such integration may facilitate more proactive, individualized maternal immunization strategies in routine obstetric care.

Nonetheless, the observed associations should be interpreted with caution due to the cross-sectional design of the study. While variables like social support or prior HPV vaccination were significantly associated with higher vaccine intention, such associations may reflect underlying vaccine confidence, healthcare-seeking behavior, or socioeconomic status, rather than true causal effects.

Despite these strengths, our study also has several limitations. First, participants were recruited solely from Guangzhou, which may limit the generalizability of our findings to other regions in China. Our use of convenience sampling from a single tertiary hospital in southern China may introduce potential selection bias. These participants, who had access to a university-affiliated maternal health center, might possess higher health literacy, better access to healthcare, and more positive vaccine attitudes compared to women in rural or socioeconomically disadvantaged settings. Thus, the generalizability of our findings may be limited. Future studies should consider multi-center sampling across diverse geographic regions and healthcare settings, including community hospitals and rural clinics, to enhance representativeness and external validity.

Second, our outcome was based on vaccine intention, which may not fully predict actual behavior. Although intention is a critical precursor to vaccination, real-world factors such as vaccine accessibility and family decision-making dynamics could influence actual vaccination choices. Moreover, due to space constraints and our focus on general vaccination willingness, the survey did not capture several potentially important predictors—such as history of preterm birth, prior vaccine adverse events, pertussis vaccination, or experience of RSV infection in older children/exposure to known RSV outbreaks. These factors may significantly influence maternal vaccination decisions and should be considered in future investigations to further improve predictive accuracy and contextual relevance.

Building upon this study, future research should explore maternal RSV vaccination willingness in other regions of China, particularly poverty-affected and remote areas. Additionally, prospective longitudinal studies should be conducted to evaluate whether maternal intention translates into actual vaccine uptake following national approval and availability.

## 5. Conclusions

This study offers the first evidence on maternal RSV vaccination willingness among pregnant and pregnancy-planning women in southern China. Willingness to vaccinate was primarily associated with younger maternal age, higher perceived social support, greater perceived risk of RSV, prior HPV vaccination, and medical insurance coverage. A predictive nomogram based on these variables showed good discrimination and calibration, providing a useful tool for identifying women with high or low intention to vaccinate. Our findings underscore the need for targeted health communication strategies, particularly those addressing age-specific concerns such as vaccine safety and neonatal protection. As maternal RSV vaccines move closer to approval in China, these insights can inform policy planning and support more effective implementation of maternal immunization programs. Future research should focus on multi-center, larger-sample studies to validate these findings, identify any suspicious influencing factors that may have led to the negative results of this study, and improve the predictive tools for wider application.

## Figures and Tables

**Table 1:** Distribution of the demographic characteristics of the participants (*n* = 406).

Characteristics	Vaccination Intention		*p*
	Yes (%) N = 273 (67.2)	No (%) N = 133 (32.8)	
Maternal Age (years)	30.00 ± 4.28	31.24 ± 4.29	0.007
Ethnicity			0.980
Han	259 (94.9)	127 (95.5)	
ethnic minorities	14 (5.1)	6 (4.5)	
Religion			0.969
No	240 (87.9)	116 (87.2)	
Yes	33 (12.1)	17 (12.8)	
Highest education			0.521
Junior high school or below	7 (2.6)	5 (3.8)	
High school or technical secondary school	18 (6.6)	10 (7.5)	
Junior College	55 (20.1)	31 (23.3)	
Bachelor’s degree	154 (56.4)	76 (57.1)	
Master’s degree or above	39 (14.3)	11 (8.3)	
Occupation			0.054
No Income or Student Stage (Student or full-time mother)	40 (14.7)	18 (13.5)	
Government or Public Sector Employee	14 (5.1)	4 (3.0)	
Professional technician	26 (9.5)	21 (15.8)	
Clerical worker	103 (37.7)	46 (34.6)	
Manager	14 (5.1)	9 (6.8)	
Worker or farmer	6 (2.2)	1 (0.8)	
Self-employed or freelancer	13 (4.8)	8 (6.0)	
Others	14 (5.1)	12 (9.0)	
Current residence			0.485
Urban	252 (92.3)	126 (94.7)	
Township	21 (7.7)	7 (5.3)	
Marital status			0.135
Unmarried	21 (7.7)	7 (5.3)	
Married	251 (91.9)	123 (92.5)	
Divorced	1 (0.4)	3 (2.3)	
Co-residents requiring care			0.229
Yes	115 (42.1)	47 (35.3)	
No	158 (57.9)	86 (64.7)	
Number of born children			0.284
≥2 children	33 (12.1)	23 (17.3)	
1 child	90 (33.0)	37 (27.8)	
None	150 (54.9)	73 (54.9)	
Pregnancy status			0.048
Preparing for pregnancy	64 (23.4)	19 (14.3)	
Early pregnancy (1–3 months)	42 (15.4)	17 (12.8)	
Mid pregnancy (4–6 months)	65 (23.8)	46 (34.6)	
Late pregnancy (7–10 months)	102 (37.4)	51 (38.3)	
Annual household income (RMB)			0.089
<50,000 RMB	22 (8.1)	20 (15.0)	
50,000–100,000 RMB	63 (23.1)	27 (20.3)	
100,000–200,000 RMB	97 (35.5)	48 (36.1)	
200,000–500,000 RMB	75 (27.5)	26 (19.5)	
>500,000 RMB	16 (5.9)	12 (9.0)	
Social support level			<0.001
Low	75 (27.5)	83 (62.4)	
High	198 (72.5)	50 (37.6)	
Support from family			<0.001
Strongly disagree	28 (10.3)	43 (32.3)	
Disagree	37 (13.6)	34 (25.6)	
Neutral	117 (42.9)	44 (33.1)	
Agree	47 (17.2)	4 (3.0)	
Strongly agree	44 (16.1)	8 (6.0)	
Support from friends			<0.001
Strongly disagree	18 (6.6)	36 (27.1)	
Disagree	35 (12.8)	36 (27.1)	
Neutral	127 (46.5)	48 (36.1)	
Agree	44 (16.1)	6 (4.5)	
Strongly agree	49 (17.9)	7 (5.3)	
Support from doctors			<0.001
Strongly disagree	20 (7.3)	29 (21.8)	
Disagree	28 (10.3)	23 (17.3)	
Neutral	118 (43.2)	65 (48.9)	
Agree	50 (18.3)	10 (7.5)	
Strongly agree	57 (20.9)	6 (4.5)	
Type of usual healthcare facility			0.687
Primary healthcare facility	64 (23.4)	22 (16.5)	
Non-primary healthcare facility	209 (76.6)	111 (83.5)	
Heard of RSV			0.023
Yes	122 (44.7)	43 (32.3)	
Never	151 (55.3)	90 (67.7)	
Information availability			0.003
Totally insufficient	73 (26.7)	62 (46.6)	
Insufficient	85 (31.1)	29 (21.8)	
Moderate	87 (31.9)	30 (22.6)	
Sufficient	19 (7.0)	8 (6.0)	
Totally sufficient	9 (3.3)	4 (3.0)	
RSV knowledge level			0.150
High level	186 (68.1)	81 (60.9)	
Low level	87 (31.9)	52 (39.1)	
Perceived RSV risk			<0.001
Low risk	98 (35.9)	77 (57.9)	
Moderate risk	131 (48.0)	40 (30.1)	
High risk	44 (16.1)	16 (12.0)	
Vaccination History			
COVID-19 vaccine			1.000
None	32 (11.7)	15 (11.3)	
Yes	241 (88.3)	118 (88.7)	
Hepatitis B vaccine			0.050
None	110 (40.3)	68 (51.1)	
Yes	163 (59.7)	65 (48.9)	
Influenza vaccine			0.730
None	211 (77.3)	100 (75.2)	
Yes	62 (22.7)	33 (24.8)	
MMR vaccine			0.783
None	221 (81.0)	108 (81.2)	
Yes	52 (19.1)	25 (18.8)	
HPV vaccine			0.019
None	133 (48.7)	82 (61.7)	
Yes	140 (51.3)	51 (38.3)	
Tetanus vaccine			0.790
None	252 (92.3)	121 (91.0)	
Yes	21 (7.7)	12 (9.0)	
Rabies vaccine			0.305
None	234 (85.7)	108 (81.2)	
Yes	39 (14.3)	25 (18.8)	
Vaccination during pregnancy			0.692
None	268 (98.2)	129 (97.0)	
Yes	5 (1.8)	4 (3.0)	
Chronic disease history			0.396
None	230 (84.2)	117 (88.0)	
Yes	43 (15.8)	16 (12.0)	
Conditions affecting vaccination			0.718
None	241 (88.3)	115 (86.5)	
Yes	32 (11.7)	18 (13.5)	
Medical insurance			0.008
Yes	266 (97.4)	121 (91.0)	
None	7 (2.6)	12 (9.0)	

Abbreviations: RSV, respiratory syncytial virus; MMR, measles–mumps–rubella; HPV, human papillomavirus; COVID-19, coronavirus disease 2019. Note: See Table S2 for detailed occupational categories.

**Table 2:** Multivariate logistic regression of vaccination intention.

Characteristics	OR (95%CI)	*p*
Maternal Age (years)	0.94 (0.89, 0.99)	0.035
Pregnancy status		
Preparing for pregnancy	reference	reference
Early pregnancy (1–3 months)	0.98 (0.39, 2.45)	0.965
Mid pregnancy (4–6 months)	0.67 (0.30, 1.48)	0.325
Late pregnancy (7–10 months)	0.99 (0.44, 2.16)	0.976
Annual household income (RMB)		
<50,000 RMB	reference	reference
50,000–100,000 RMB	1.89 (0.75, 4.80)	0.175
100,000–200,000 RMB	1.37 (0.58, 3.22)	0.473
200,000–500,000 RMB	2.25 (0.89, 5.66)	0.084
>500,000 RMB	0.98 (0.31, 3.07)	0.968
Social support level		
Low	reference	reference
High	3.85 (2.33, 6.46)	<0.001
Heard of RSV		
Never	reference	reference
Yes	1.33 (0.75, 2.36)	0.327
Information availability		
Totally insufficient	reference	reference
Insufficient	1.72 (0.92, 3.22)	0.088
Moderate	1.33 (0.69, 2.59)	0.398
Sufficient	0.48 (0.16, 1.51)	0.200
Totally sufficient	0.91 (0.22, 4.07)	0.893
RSV knowledge level		
Low level	reference	reference
High level	0.86 (0.51, 1.44)	0.570
Perceived RSV risk		
Low risk	reference	reference
Moderate risk	2.23 (1.32, 3.81)	0.003
High risk	2.12 (1.03, 4.51)	0.045
Hepatitis B vaccine		
None	reference	reference
Yes	1.17 (0.71, 1.91)	0.537
HPV vaccine		
None	reference	reference
Yes	1.77 (1.07, 2.95)	0.026
Medical insurance		
None	reference	reference
Yes	3.77 (1.17, 12.86)	0.028

Abbreviations: RSV, respiratory syncytial virus; HPV, human papillomavirus.

## Data Availability

The data presented in this study are not publicly available due to privacy and ethical restrictions. The questionnaire data contain personal and sensitive information from study participants and cannot be shared publicly.

## Supplementary Materials

The following supporting information can be downloaded at: https://www.mdpi.com/article/10.3390/vaccines14020160/s1. Figure S1: Receiver operating characteristic (ROC) curve of the nomogram based on multivariable logistic regression, showing good discrimination (AUC = 0.753); Figure S2: Calibration plot using 1000 bootstrap resamples. The model predictions closely matched observed probabilities (mean absolute error = 0.014); Figure S3: Decision curve analysis (DCA) illustrating the net benefit of the nomogram compared to treat-all or treat-none strategies; Table S1: Definition, measurement, and coding of variables included in the analysis; Table S2: Distribution of the detailed occupational categories; Table S3: The univariable analysis of transfer rate and neonatal IgG antibody titers; Table S4: Nomogram Scoring Table; Table S5: Sensitivity analysis using continuous versions of social support, perceived risk, and RSV knowledge score in multivariable logistic regression predicting willingness to receive maternal RSV vaccination.

## Abbreviations

The following abbreviations are used in this manuscript:

RSV	Respiratory Syncytial Virus
IgG	Immunoglobulin G
pre-F	Pre-fusion conformation
GMT	Geometric Mean Titer
ELISA	Enzyme-Linked Immunosorbent Assay
OR	Odds Ratio
CI	Confidence Interval
AUC	Area Under the Curve
ROC	Receiver Operating Characteristic
SD	Standard Deviation
IQR	Interquartile Range
WHO	World Health Organization
HPV	Human Papillomavirus
COVID-19	Coronavirus Disease 2019
SARS-CoV-2	Severe Acute Respiratory Syndrome Coronavirus 2
