# Supplementary material for: Willingness to Receive Maternal RSV Vaccination Among Pregnant Women and Those Planning Pregnancy in Southern China: A Cross-Sectional Study and Predictive Nomogram
Source: Vaccines (Basel). 2026 Feb 8;14(2):160. doi: 10.3390/vaccines14020160 (PMC12945216; doi:10.3390/vaccines14020160)
Supplement: Supplementary file 1 [file vaccines-14-00160-s001.zip › vaccines-4117967-supplemental questionnaire.pdf]

Survey on Maternal RSV Vaccine Acceptance Among Preconception and Pregnant Women

Dear participant,

*We are a research team from the School of Public Health at Sun Yat-sen University conducting a survey on the willingness to receive vaccination against respiratory syncytial virus (RSV). RSV is the leading cause of acute respiratory infections in **infants and young children** and one of the primary causes of related deaths.*

*The target population of this survey is **women aged 18–49 who are either currently pregnant or preparing for pregnancy**. No personal information will be disclosed, and all responses will be kept strictly confidential and used for research purposes only. Thank you for your support and cooperation!*

Section 1: Predisposing Factors

1. **Gender:** □ Male (End of answer) □ Female
2. **Your age**: ______ years
3. **What is your ethnicity?** _______________
4. **Do you have any religious belief?**
   □ Yes (e.g., Buddhism, Christianity, Taoism, Islam…) □ No
5. **What is your highest level of education?**
   □ Junior high school or below □ High school/technical secondary school
   □ Junior college □ Bachelor's degree □ Master's degree or above
6. **What is your current occupation?**
   □ Student □ Full-time mother □ Civil servant □ Professional technician
   □ Clerk □ Corporate manager □ Worker or farmer □ Active military personnel □ Self-employed □ Freelancer □ Other (please specify): ______
7. **Where do you currently live?** Province: ______ City: ______
8. **Type of current residence:**
   □ Urban □ Township
9. **What is your marital status?**
   □ Unmarried □ Married □ Divorced □ Widowed
10. **Do any of your co-residents** (excluding yourself) **belong to the following groups?** (Check all that apply)
    □ Pregnant women □ Children (under 18) □ Elderly (60 years or older)
    □ People with chronic diseases (e.g., hypertension, diabetes)
    □ Others requiring special care (please specify): __________
    □ None of the above

11. Do you currently have any children? (Please do not count your current pregnancy)

□ More than one child □ One child □ None

12. What is your current pregnancy status?

□ 1st trimester (1–3 months) □ 2nd trimester (4–6 months)

□ 3rd trimester (7–10 months) □ Preparing for pregnancy

Section 2: Enabling Resources

13. What is your annual household income (RMB)?

□ <50000 □ 50000–100000 □ 100000–200000 □ 200000–500000 □ >500,000

14. Are you registered as a low-income household?

□ Yes □ No

15. ***Please indicate your level of agreement with the following statements:***

15-1. My family members will support my vaccination during pregnancy.

□ Strongly disagree □ Disagree □ Neutral □ Agree □ Strongly agree

15-2. My friends will support my vaccination during pregnancy.

□ Strongly disagree □ Disagree □ Neutral □ Agree □ Strongly agree

15-3. Doctors or healthcare professionals recommend vaccination during pregnancy and explains its benefits.

□ Strongly disagree □ Disagree □ Neutral □ Agree □ Strongly agree

16. What type of medical facility do you usually visit?

□ Outpatient clinic □ Village clinic □ Community health station

□ Community health center □ Township hospital □ County/district hospital

□ Municipal hospital □ Provincial hospital or above □ Private hospital

Section 3: Health Behaviors and Awareness

17. Have you heard of respiratory syncytial virus (RSV)? If yes, through which sources? (Check all that apply)

□ Medical personnel □ Internet and social media □ Institutions/organizations

□ Family or friends □ Scientific journals □ Others □ Never heard of

18. Do you think the available information on RSV is sufficient?

□ Totally insufficient □ Insufficient □ Moderate □ Sufficient □ Totally sufficient

19–24. Please indicate whether the following statements are true or false:

19. Both children and adults infected with RSV can serve as sources of infection.

□ True □ False

20. RSV is mainly transmitted through droplets (such as coughing and sneezing) and direct contact.

□ True □ False

21. Newborns and infants are susceptible to RSV.

□ True □ False

22. Typical symptoms of RSV infection include cough, shortness of breath, and fever.

□ True □ False

23. RSV infection has no incubation period.

□ True □ False

24. Maintaining hand hygiene and avoiding contact with infected people are effective measures to prevent the spread of RSV.

□ True □ False

25–27. Please rate your perception of RSV risk:

25. I think RSV is a serious infectious disease.

□ Strongly disagree □ Disagree □ Neutral □ Agree □ Strongly agree

26. I am concerned that RSV could spread widely in China.

□ Strongly disagree □ Disagree □ Neutral □ Agree □ Strongly agree

27. I am worried that I or my family members (including my baby) may be infected with RSV.

□ Strongly disagree □ Disagree □ Neutral □ Agree □ Strongly agree

28. Have you received any of the following vaccines? (Check all that apply)

□ COVID-19 □ Hepatitis B □ Influenza □ MMR □ HPV □ Tetanus

□ Rabies □ Other (please specify): ______ □ Never vaccinated

29. Were any of the above vaccines received during pregnancy?

□ Yes □ No

Section 4: Need Factors

30. Do you have any of the following chronic diseases?

□ None □ Hypertension □ Diabetes □ Chronic digestive disorders

□ Cardiovascular disease or stroke □ ENT chronic conditions

□ Chronic respiratory disease □ Other (please specify): _______

31. Have you ever had any of the following health conditions or experiences that might affect your eligibility for RSV vaccination? (Check all that apply)

□ Allergy to vaccine components (e.g., aluminum, thiomersal)

□ Severe allergic reaction to any vaccine or injection (e.g., anaphylactic shock, breathing difficulty)

□ Immunocompromised status or immunosuppressive therapy (e.g., chemotherapy, organ transplant medication)

□ Recent receipt of blood products or immunoglobulin

□ None of the above

32. Have you participated in any of the following medical insurance programs? (Multiple selections allowed)

□ Social medical insurance (including basic medical insurance for urban employees, basic medical insurance for urban and rural residents, public medical insurance, and other social insurance programs)

□ Supplementary medical insurance (critical illness insurance, long-term care insurance, etc.)

□ Commercial medical insurance

□ None of the above

33. Although RSV vaccines are not currently approved for pregnant women in China, which of the following factors might influence your future decision to receive one if it becomes available? (Check all that apply)

□ Price and reimbursement coverage

□ Convenience of vaccination site (e.g., distance, transportation)

□ Speed and comfort of the vaccination process

□ Vaccine safety (e.g., risk of adverse effects)

□ Protective effect on the newborn

34. If recommended by your doctor or national guidelines, would you be willing to receive an RSV vaccine between 24 and 36 weeks of pregnancy to protect your newborn?

□ Willing □ Unwilling

Thank you for your participation!

Your responses will help us better understand the attitudes and needs of pregnant and preconception women regarding RSV vaccination, contributing to more evidence-based public health strategies.
