# Supplementary material for: Willingness to Receive Maternal RSV Vaccination Among Pregnant Women and Those Planning Pregnancy in Southern China: A Cross-Sectional Study and Predictive Nomogram
Source: Vaccines (Basel). 2026 Feb 8;14(2):160. doi: 10.3390/vaccines14020160 (PMC12945216; doi:10.3390/vaccines14020160)
Supplement: Supplementary file 1 [file vaccines-14-00160-s001.zip › vaccines-4117967-supplementary tables and figures.pdf]

Table S1. Definition, measurement, and coding of variables included in the analysis.

Domain	Variable	Question	Measurement	Categories / Scale
Predisposing	Maternal age	Q2	Continuous	Years
	Ethnicity	Q3	Categorical	Han / Ethnic minorities
	Religion	Q4	Binary	No / Yes
	Highest education	Q5	Categorical	Junior high school or below;<br>High school/technical secondary school;<br>Junior college;<br>Bachelor's degree;<br>Master's degree or above
	Occupation	Q6	Categorical	No Income or Student Stage (Student or full-time mother);<br>Government or Public Sector Employee;<br>Professional technician;<br>Clerical worker;<br>Manager;<br>Worker or farmer;<br>Self-employed or freelancer;<br>Others
	Current residence	Q8	Binary	Urban / Township
	Marital status	Q9	Categorical	Unmarried;<br>Married;<br>Divorced
	Co-residents requiring care	Q10	Binary	Yes / No
	Number of born children	Q11	Categorical	⩾2 children;<br>1 child;<br>None
	Pregnancy status	Q12	Categorical	Preparing for pregnancy;<br>Early pregnancy (1–3 months);<br>Mid pregnancy (4–6 months);<br>Late pregnancy (7–10 months)
Enabling	Annual household income (RMB)	Q13	Categorical	< 50000 RMB;<br>50000-100000 RMB;<br>100000-200000 RMB;<br>200000-500000 RMB;<br>> 500000 RMB
	Social support level	Q15	Binary (derived)	Low / High (Likert-based)
	Support from family	Q15-1	Likert (5-point)	Strongly disagree → Strongly agree
	Support from friends	Q15-2		
	Support from doctors	Q15-3		
	Type of usual healthcare facility	Q16	Binary	Primary / Non-primary
	Medical insurance	Q32	Binary	Yes / None
Health behavior	Heard of RSV	Q17	Binary	Yes / Never
	Information availability	Q18	Ordinal	Totally insufficient → Totally sufficient
	RSV knowledge level	Q19–24	Binary (score)	High / Low (median cut)
	Perceived RSV risk	Q25–27	Ordinal	Low / Moderate / High
	COVID-19 vaccination<br>Hepatitis B vaccination<br>Influenza vaccination<br>MMR vaccination<br>HPV vaccination<br>Tetanus vaccination<br>Rabies vaccination	Q28	Binary	Yes / None
	Vaccination during pregnancy	Q29	Binary	Yes / None

Need	Chronic disease history	Q30	Binary	Yes / None
	Conditions affecting vaccination	Q31	Binary	Yes / None

Table S2. Distribution of the detailed occupational categories.

Characteristics	Vaccination Intention		*p*
	**Yes (%)** *N* **= 273 (67.2)**	**No (%)** *N* **= 133 (32.8)**	
**Occupation**			0.054
No Income or Student Stage (Student or full-time mother)	40 (14.7)	18 (13.5)	
Government or Public Sector Employee	14 (5.1)	4 (3.0)	
Professional technician	26 (9.5)	21 (15.8)	
Clerical worker	103 (37.7)	46 (34.6)	
Manager	14 (5.1)	9 (6.8)	
Worker or farmer	6 (2.2)	1 (0.8)	
Self-employed or freelancer	13 (4.8)	8 (6.0)	
Others	14 (5.1)	12 (9.0)	

Table S3. Univariable logistic regression analysis of factors associated with willingness to receive maternal RSV vaccination.

Characteristics	OR (95%CI)	*p*
**Maternal Age (years)**	0.94 (0.89, 0.98)	0.007
**Ethnicity**		
Han	reference	reference
ethnic minorities	1.29 (0.37, 5.96)	0.706
**Religion**		
No	reference	reference
Yes	0.67 (0.27, 1.76)	0.392
**Highest education**		
Junior high school or below	reference	reference
High school or technical secondary school	1.29 (0.31, 5.15)	0.722
Junior College	1.27 (0.35, 4.31)	0.706
Bachelor's degree	1.45 (0.42, 4.68)	0.539
Master's degree or above	2.53 (0.64, 9.61)	0.170
**Occupation**		
No Income or Student Stage (Student or full-time mother)	reference	reference
Government or Public Sector Employee	1.01 (0.52, 1.92)	0.982
Professional technician	1.57 (0.48, 6.15)	0.474
Clerical worker	0.56 (0.25, 1.24)	0.152
Manager	0.70 (0.26, 1.95)	0.487
Worker or farmer	2.70 (0.42, 52.96)	0.374
Self-employed or freelancer	1.15 (0.54, 2.41)	0.720
Others	0.53 (0.20, 1.37)	0.184
**Current residence**		
Urban	reference	reference
Township	1.50 (0.65, 3.89)	0.367
**Marital status**		
Married	reference	reference
Unmarried	1.47 (0.64, 3.82)	0.392
Divorced	0.16 (0.01, 1.29)	0.118
**Co-residents requiring care**		
No	reference	reference
Yes	1.33 (0.87, 2.04)	0.191
**Number of born children**		
None	reference	reference
1 child	1.18 (0.74, 1.91)	0.242
≥ 2 children	0.70 (0.38, 1.28)	0.485
**Pregnancy status**		
Preparing for pregnancy	reference	reference
Early pregnancy (1–3 months)	0.73 (0.34, 1.58)	0.425
Mid pregnancy (4–6 months)	0.42 (0.22, 0.78)	0.007
Late pregnancy (7–10 months)	0.59 (0.32, 1.08)	0.095
**Annual household income (RMB)**		
< 50000 RMB	reference	reference
50000-100000 RMB	2.12 (1.00, 4.54)	0.051
100000-200000 RMB	1.84 (0.91, 3.70)	0.087
200000-500000 RMB	2.62 (1.24, 5.60)	0.012
> 500000 RMB	1.21 (0.46, 3.21)	0.695
**Social support level**		
Low	reference	reference
High	4.38 (2.84, 6.84)	< 0.001
**Type of usual healthcare facility**		
Primary healthcare facility	reference	reference
Non-primary healthcare facility	0.65 (0.37, 1.09)	0.112
**Heard of RSV**		
Never	reference	reference
Yes	1.69 (1.10, 2.63)	0.018

**Information availability**		
Totally insufficient		
Insufficient	2.49 (1.46, 4.32)	0.001
Moderate	2.46 (1.45, 4.25)	0.001
Sufficient	2.02 (0.85, 5.18)	0.123
Totally sufficient	1.91 (0.59, 7.33)	0.300
**RSV knowledge level**		
Low level	reference	reference
High level	0.73 (0.47, 1.12)	0.150
**Perceived RSV risk**		
Low risk	reference	reference
Moderate risk	2.57 (1.63, 4.12)	< 0.001
High risk	2.16 (1.15, 4.22)	0.019
**Vaccination History**		
COVID-19 vaccine (none as reference)	1.04 (0.55, 2.05)	0.896
Hepatitis B vaccine (none as reference)	0.65 (0.42, 0.98)	0.039
Influenza vaccine (none as reference)	1.12 (0.69, 1.81)	0.639
MMR vaccine (none as reference)	1.00 (0.58, 1.69)	0.991
HPV vaccine (none as reference)	0.59 (0.39, 0.90)	0.015
Tetanus vaccine (none as reference)	1.19 (0.55, 2.46)	0.646
Rabies vaccine (none as reference)	1.39 (0.79, 2.40)	0.243
**Vaccination during pregnancy**		
None	reference	reference
Yes	1.66 (0.41, 6.38)	0.455
**Chronic disease history**		
None	reference	reference
Yes	1.37 (0.75, 2.60)	0.319
**Conditions affecting vaccination**		
None	reference	reference
Yes	0.85 (0.46, 1.60)	0.602
**Medical insurance**		
None	reference	reference
Yes	3.77 (1.48, 10.35)	0.007

Table S4. Nomogram Scoring Table.

Variable	Level	Linear Predictor (X_β)	Points
Maternal age (years)	18	1.147	100.00
Maternal age (years)	20	1.274	92.31
Maternal age (years)	22	1.402	84.62
Maternal age (years)	24	1.529	76.92
Maternal age (years)	26	1.657	69.23
Maternal age (years)	28	1.784	61.54
Maternal age (years)	30	1.911	53.85
Maternal age (years)	32	2.039	46.15
Maternal age (years)	34	2.166	38.46
Maternal age (years)	36	2.294	30.77
Maternal age (years)	38	2.421	23.08
Maternal age (years)	40	2.549	15.38
Maternal age (years)	42	2.676	7.69
Maternal age (years)	44	2.803	0.00
Social support level	Low	0.000	0.00
Social support level	High	1.353	81.69
Knowledge level	Low	0.000	1.52
Knowledge level	High	0.025	0.00
Perceived RSV risk	Low	0.000	0.00
Perceived RSV risk	Moderate	0.768	46.35
Perceived RSV risk	High	0.562	33.89
HPV vaccine	None	0.000	0.00
HPV vaccine	Yes	0.160	9.68
Medical insurance	None	0.000	0.00
Medical insurance	Yes	0.535	32.28

Table S5. Sensitivity analysis using continuous versions of social support, perceived risk, and RSV knowledge score in multivariable logistic regression predicting willingness to receive maternal RSV vaccination.

Characteristics	β / OR (95%CI)	p
**Maternal Age (years)**	-0.06 (-0.13, -0.01)	0.032
**Pregnancy status**		
Preparing for pregnancy	reference	reference
Early pregnancy (1–3 months)	0.98 (0.39, 2.47)	0.962
Mid pregnancy (4–6 months)	0.73 (0.32, 1.63)	0.447
Late pregnancy (7–10 months)	1.12 (0.50, 2.46)	0.780
**Annual household income (RMB)**		
< 50000 RMB	reference	reference
50000-100000 RMB	1.76(0.68, 4.55)	0.239
100000-200000 RMB	1.30 (0.54, 3.12)	0.554
200000-500000 RMB	1.85 (0.72, 4.69)	0.195
> 500000 RMB	0.75 (0.23, 2.42)	0.632
**Social support level**	1.32 (1.21, 1.46)	<0.001
**Heard of RSV**		
Never	reference	reference
Yes	1.32 (0.75, 2.33)	0.345
**Information availability**		
Totally insufficient	reference	reference
Insufficient	1.68 (0.91, 3.15)	0.100
Moderate	1.31 (0.67, 2.54)	0.428
Sufficient	0.34 (0.11, 1.12)	0.073
Totally sufficient	0.53 (0.12, 2.62)	0.415
**RSV knowledge level**	0.91 (0.62, 1.31)	0.616
**Perceived RSV risk**	1.12 (1.00, 1.25)	0.044
**Hepatitis B vaccine**		
None	reference	reference
Yes	1.13 (0.68, 1.86)	0.631
**HPV vaccine**		
None	reference	reference
Yes	1.71 (1.03, 2.85)	0.039
**Medical insurance**		
None	reference	reference
Yes	3.40 (1.03, 12.21)	0.042

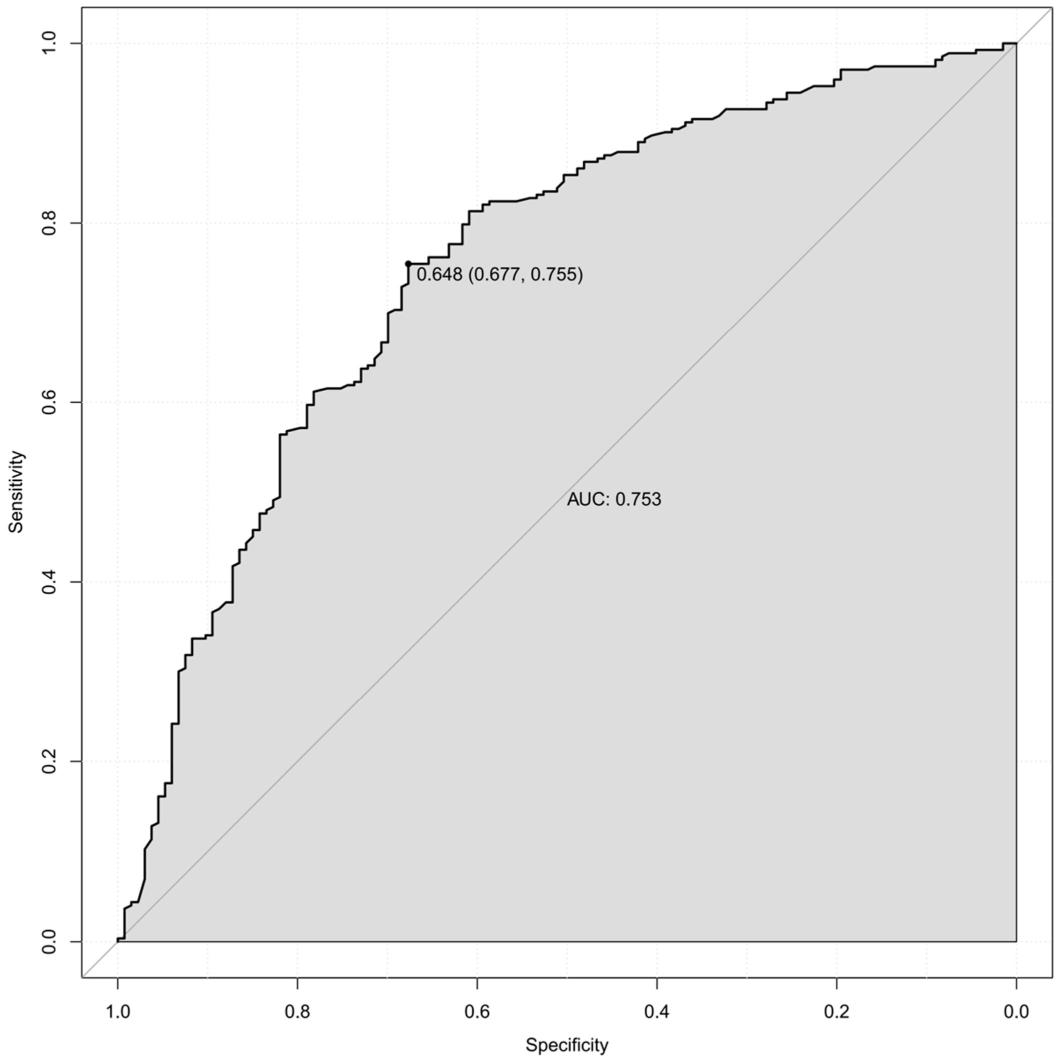

Figure S1. Receiver operating characteristic (ROC) curve of the nomogram based on multivariable logistic regression, showing good discrimination (AUC = 0.753).

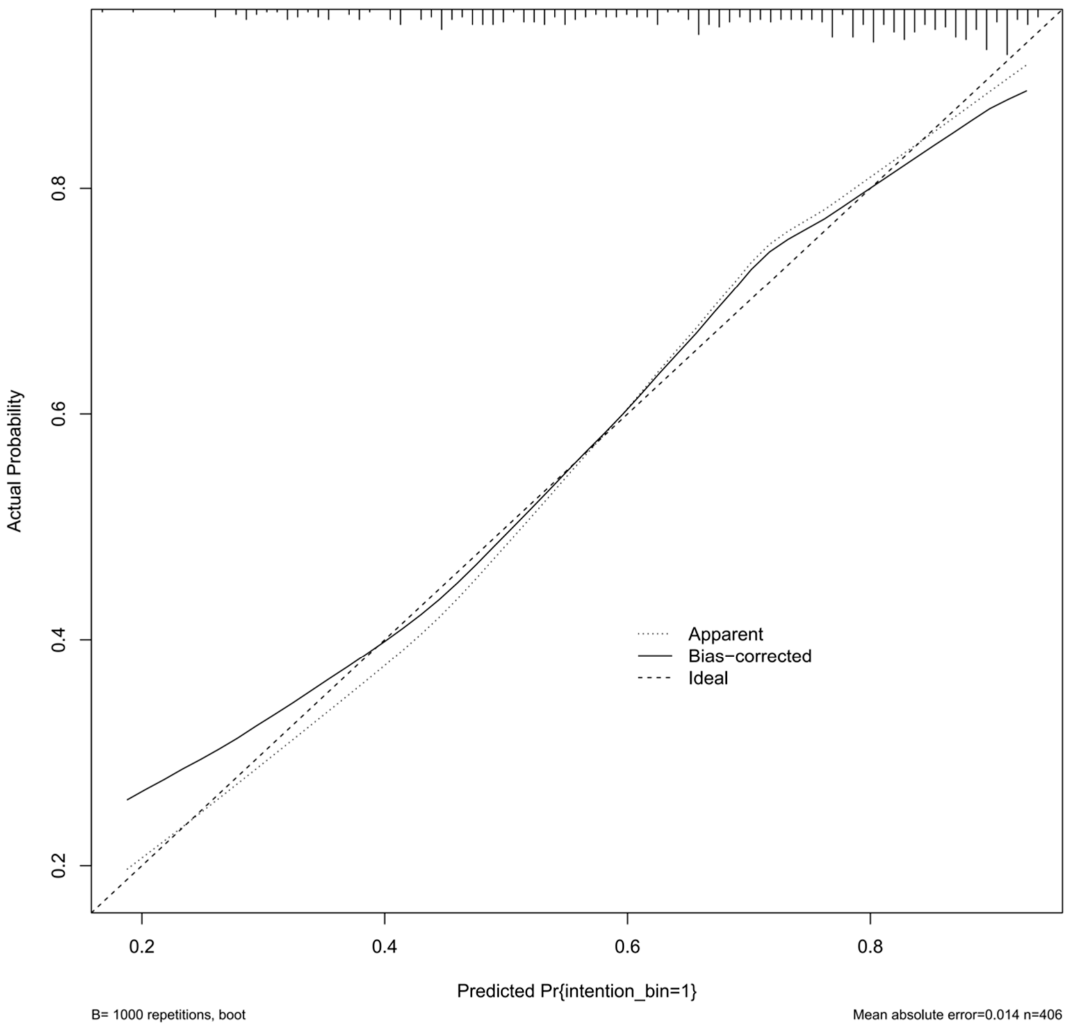

Figure S2. Calibration plot using 1000 bootstrap resamples. The model predictions closely matched observed probabilities (mean absolute error = 0.014).

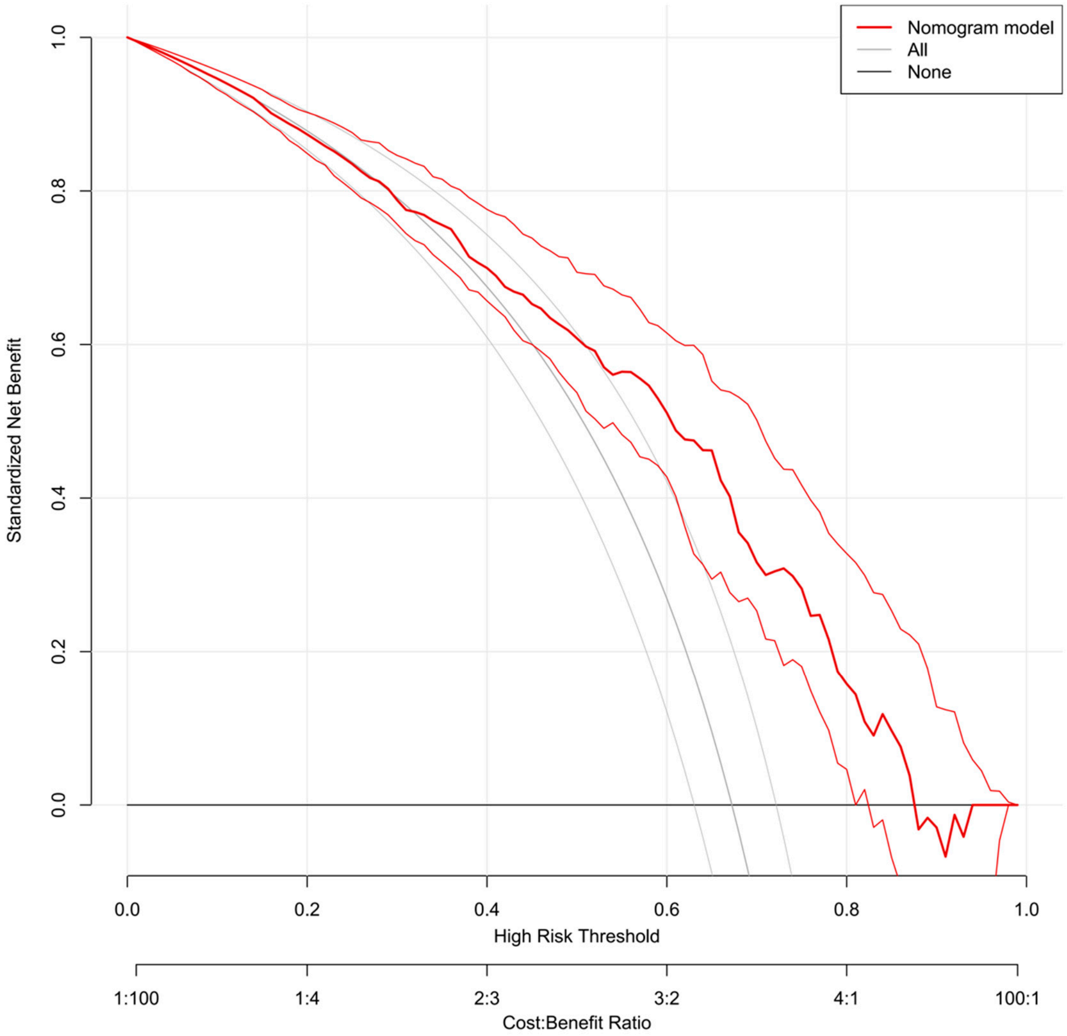

Figure S3. Decision curve analysis (DCA) illustrating the net benefit of the nomogram compared to treat-all or treat-none strategies.
